# Supplementary figures and images for: Unravelling tumour cell diversity and prognostic signatures in cutaneous melanoma through machine learning analysis
Source: J Cell Mol Med. 2024 Jul 25;28(14):e18570. doi: 10.1111/jcmm.18570 (PMC11272603; doi:10.1111/jcmm.18570)

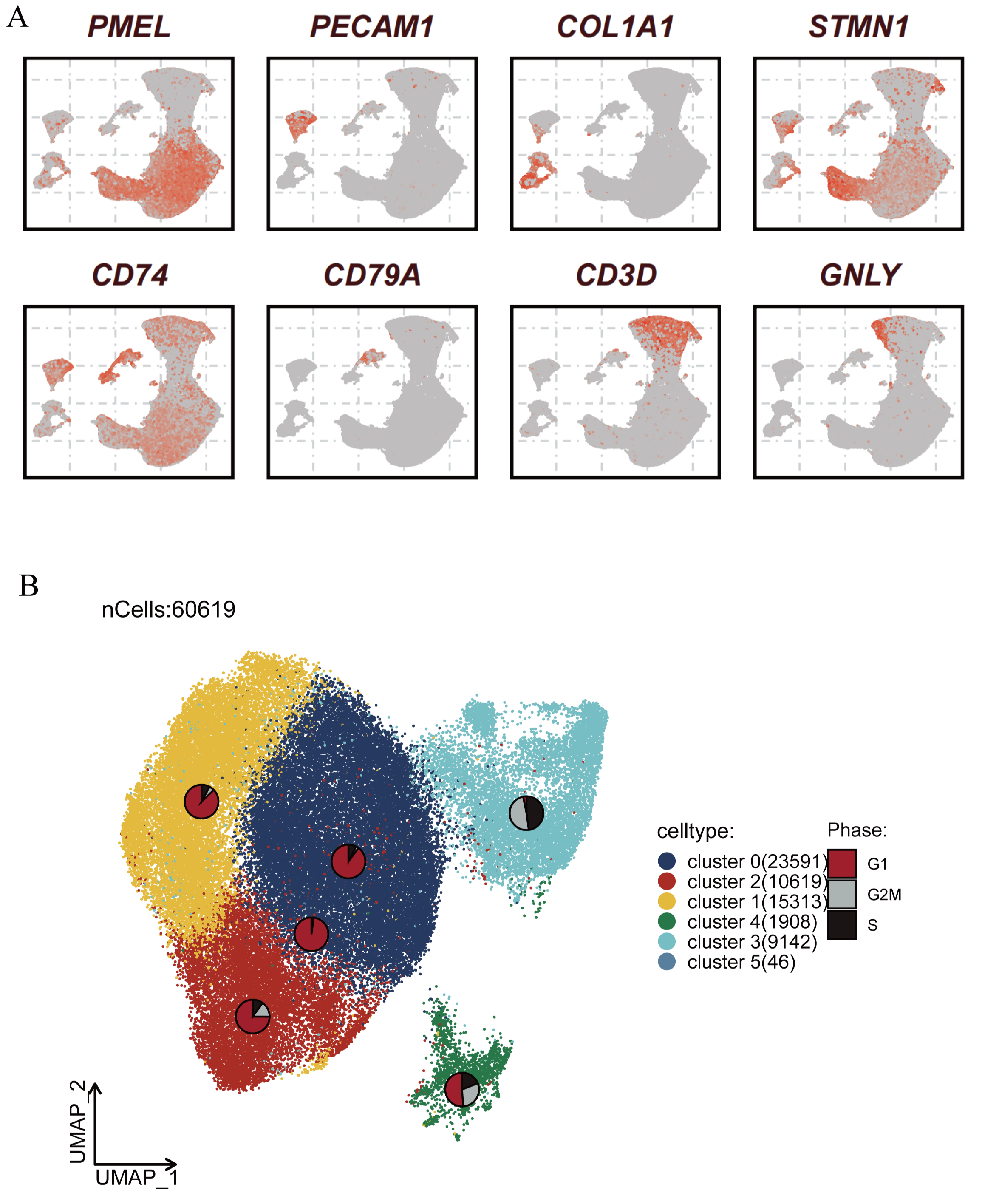

Supplement: Supplementary file 1 — Figure S1. UMAP Visualization of Cell Type Markers and Cell Cycle States (A) UMAP plot showing the expression levels of classical cell type marker genes across different clusters. (B) UMAP plot displaying the distribution of different tumour clusters, with pie charts indicating the cell cycle status of each cluster. [file JCMM-28-e18570-s002.tif]

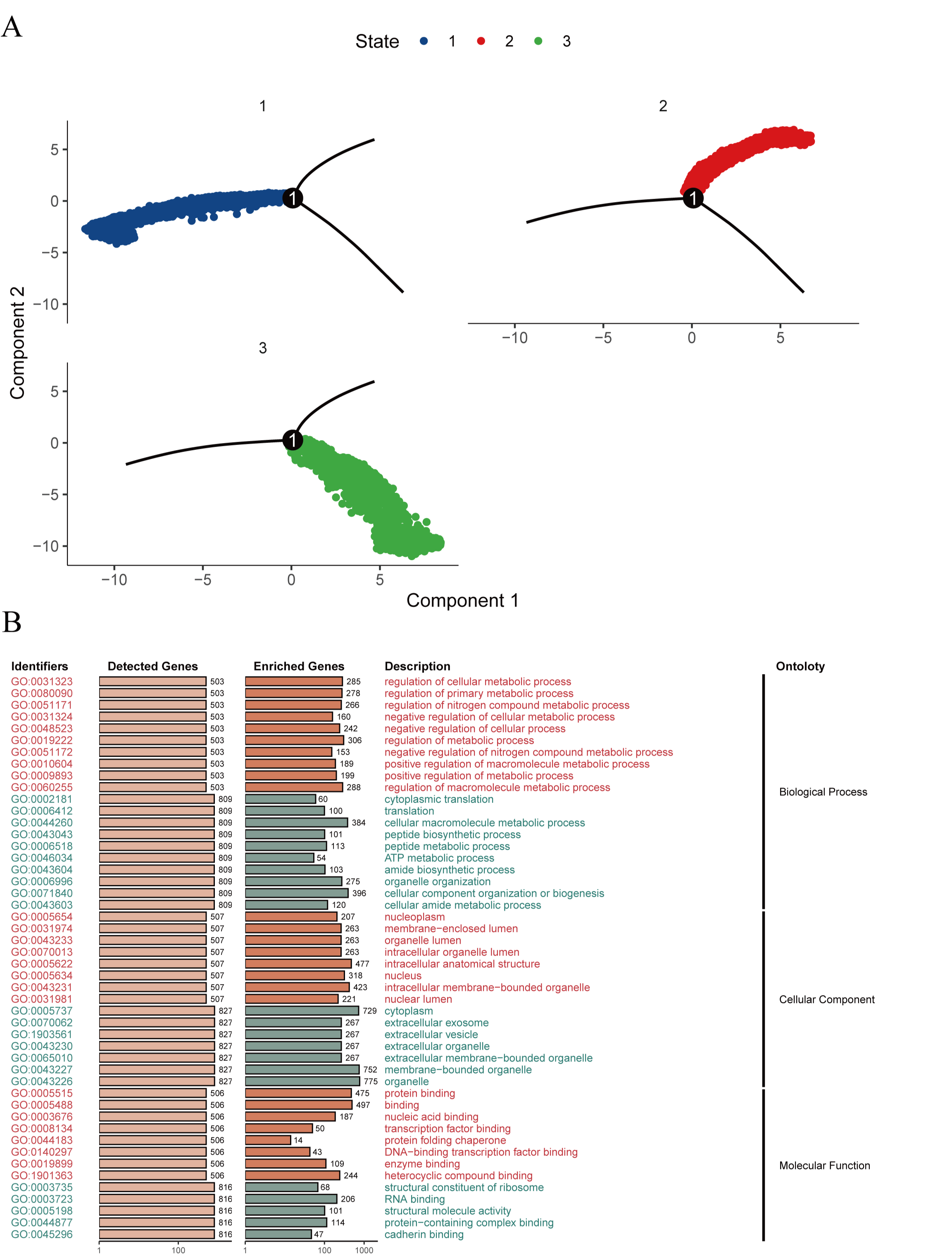

Supplement: Supplementary file 2 — Figure S2. Pseudotime Analysis of Tumour Cell Clusters and Gene Ontology Enrichment (A) Pseudotime analysis showing three distinct branches of tumour cell clusters. (B) Gene Ontology (GO) enrichment analysis of genes associated with pseudotime. [file JCMM-28-e18570-s003.tif]

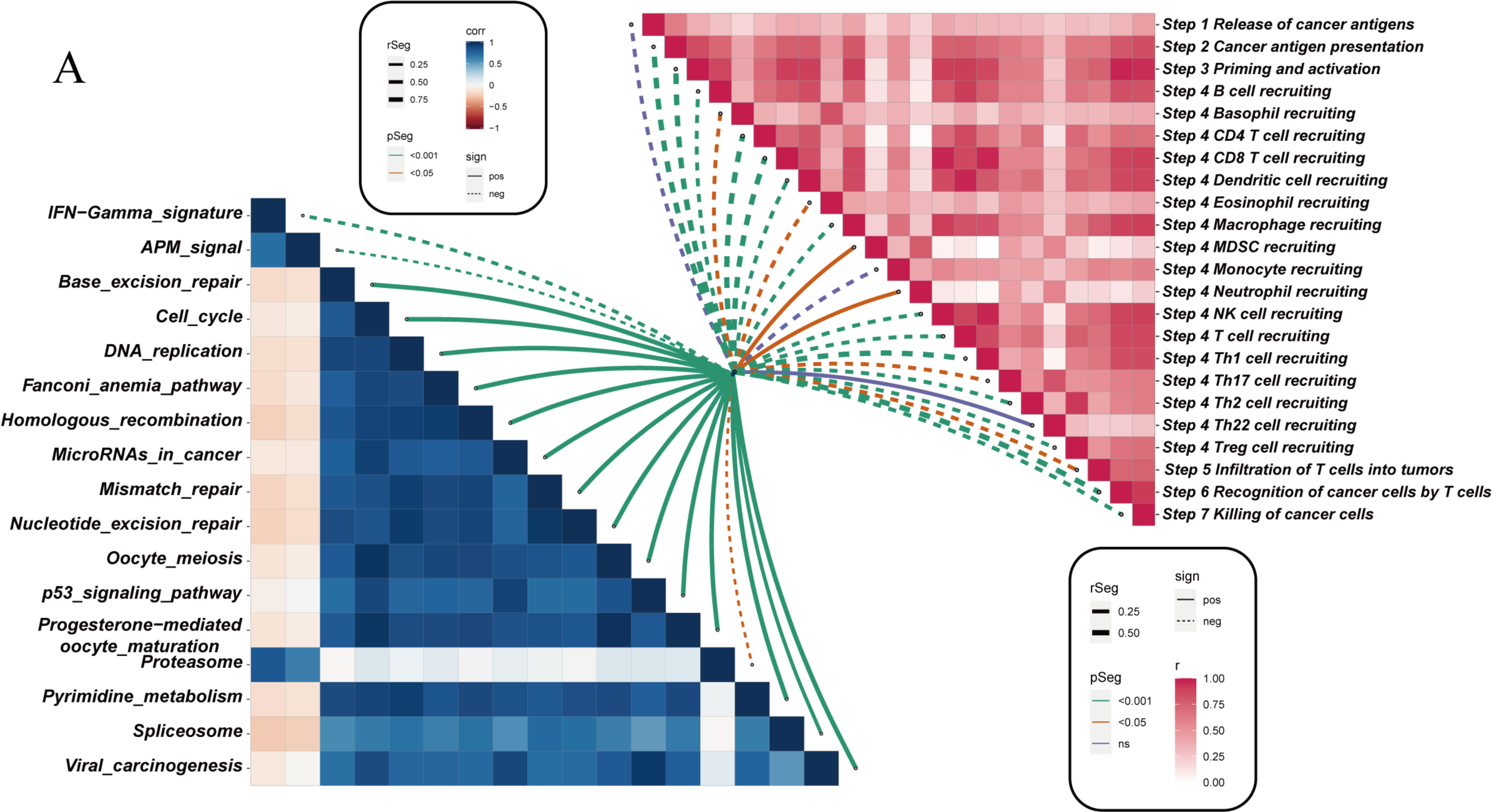

Supplement: Supplementary file 3 — Figure S3. Utilizing GSVA to evaluate the correlation between TCM scores and pathways related to immune therapy and the immune cycle. [file JCMM-28-e18570-s004.tif]
