# Supplementary material for: Unravelling tumour cell diversity and prognostic signatures in cutaneous melanoma through machine learning analysis
Source: J Cell Mol Med. 2024 Jul 25;28(14):e18570. doi: 10.1111/jcmm.18570 (PMC11272603; doi:10.1111/jcmm.18570)
Supplement: Supplementary file 4 — Table S1. Oligonucleotides used in research. [file JCMM-28-e18570-s001.docx]

| **Oligonucleotides** | **Nucleotide sequence (5'-3')** |
| --- | --- |
| **siRNA** |  |
| Si-TOMM40-1 | CAAAGGGTTGAGTAACCATTT |
| Si-TOMM40-2 | CATGTCTCTAGCTGGGAAATA |
|  |  |
| **Primer** |  |
| GAPDH | GGCCTCCAAGGAGTAAGACC (forward) |
|  | AGGGGAGATTCAGTGTGGTG (reverse) |
| TOMM40 | ACCATGGGGAACGTGTTGG (forward) |
|  | GTCCGTTCCGAACTTCGACT (reverse) |
|  |  |

**Table S1. Oligonucleotides used in research**
